# A Study on Circular Economy Material Using Fish Scales as a Natural Flame Retardant and the Properties of Its Composite Materials

**DOI:** 10.3390/polym13152446

**Published:** 2021-07-25

**Authors:** Shang-Hao Liu, Ming-Yuan Shen, Cheng-You Yang, Chin-Lung Chiang

**Affiliations:** 1 Department of Ammunition Engineering and Explosion Technology, Anhui University of Science and Technology, Huainan 232001, China; u9414042@cmu.edu.tw; 2 Department of Mechanical Engineering, National Chin-Yi University of Technology, Taichung 41170, Taiwan; 3 Green Flame Retardant Material Research Laboratory, Department of Safety, Health and Environmental Engineering, Hung-Kuang University, Taichung 433, Taiwan; willian2546jane@gmail.com

**Keywords:** fishery waste, epoxy, fish scale, ammonium polyphosphate, flame retardant

## Abstract

Fish scales (FSs) are fishery wastes that can cause environmental pollution. This study aimed to solve this environmental problem. FSs were used as a flame retardant for polymer materials, making them valuable. Fish scales were combined with a commercial flame retardant, ammonium polyphosphate (APP), through synergistic effects to reduce the amount of commercial flame retardant. The use of FSs conforms to the concept of a circular economy and lowers costs by reducing the consumption of APP. Thermogravimetric analysis (TGA), integral procedural decomposition temperature (IPDT), pyrolysis kinetics, limiting oxygen index (LOI), the Underwriters Laboratories 94 (UL94) flammability test, scanning election microscopy, Raman spectroscopy, and energy-dispersive X-ray spectroscopy were used to determine the thermal properties, flame retardant properties, flame retardant mechanism, char morphology, and composition of the composites. The TGA results indicated that the addition of 40% flame retardant raised the char residue from 16.45 wt.% (pure EP) to 36.07 wt.%; IPDT from 685.6 °C (pure EP) to 1143.1°C; LOI from 21% (pure EP) to 30%; and UL94 classification from fail (pure EP) to V-0. These results suggest an increase in char residue, which indicates better protection of the polymer matrix material. The improvements in IPDT, LOI, and UL94 classification, which indicate greater thermal stability, lower flammability (from flammable to fireproof), and higher flammability rating (from fail to V-0), respectively, suggest that the composite material has favorable thermal properties and is less inflammable.

## 1. Introduction

Epoxy resin (EP) has been used in various fields, such as aeronautics, chemistry, civil engineering, automobiles, recreation, electronics, and shipbuilding. It is used as a material for circuit boards, movable parts, coatings, and numerous other applications because of its excellent insulating and chemical-resistant properties as well as its minimal cure shrinkage. However, EP has problems such as high flammability, low impact resistance, and poor weather resistance, which prevent it from having a wider range of applications [1,2,3,4].

Several flame retardant compounds have been used to enhance the flame retardancy properties of polymeric materials [5]. Traditionally, this can be achieved by combining them with halogen-based additives. However, many potential applications of halogen-based flame retardants are prohibited due to their hazardous effects on health and the environment [6,7,8]. Therefore, several studies have been performed on halogen-free flame retardants such as metal hydroxides, phosphorus, boron-containing compounds and inorganic fillers [9,10,11]. Halogen-free flame retardants have become mainstream in applications and research.

EP is highly flammable, and when it burns, it releases a large amount of smoke and volatile gases. Thus, the presence of EP in a fire can make egress difficult and lead to serious casualties. For this reason, this study proposed introducing a composite fish scale (FS)–phosphorus flame retardant to make EP less inflammable. Many studies have specified that fish or domestic waste possess potential applications in various industrial sectors. Xia et al. reported that FSs enhanced the adhesion, viscoelastic properties, temperature susceptibility, and resistance to the permanent deformation of asphalt. It is promising and feasible to use FSs as a bio-modifier for asphalt binders and reduce waste treatment costs [12]. Dordevic’s teams developed the utilization of waste frying oils in home-made soap production. The results indicated that soaps produced from fried plant oils represent acceptable products from economic and environmental points of view [13]. Tremlova et al. also found out the obtained results did not show exact differences between experimentally produced soap samples from fried or unfried oils [14]

FSs were chosen as an additive because Taiwan is an island surrounded by sea with a prosperous fishing industry, but has little use for FSs. Most of the FSs removed from fish are discarded, and only small portions are used for compost, collagen extraction, and corneal cell culture. FSs consist of collagen and hydroxyapatite (Ca₅(PO₄)₃) [15]. Hydroxyapatite contains phosphorus and calcium elements, which accelerate the formation of a char layer and increase char yield (CY). Collagen contains nitrogen, which releases large amounts of nonflammable gases when burnt that lower the concentration of oxygen in the air. These properties allow FSs to effectively reduce the inflammability of a material; therefore, it is a type of natural flame retardant.

The utilization of FSs in halogen-free flame retardants to recycle this sustainable material has not been reported in the existing literature. This study developed a composite material that combined EP with a flame retardant to reduce the inflammability of EP. The flame retardant consisted of a flame retardant agent, ammonium polyphosphate (APP), which contains highly incombustible phosphorus and nitrogen, and FSs. The composite material was tested using thermogravimetric analysis (TGA), limiting oxygen index (LOI), the Underwriters Laboratories 94 (UL-94) flammability test, scanning election microscopy (SEM), and Raman spectroscopy to determine its thermal properties, flame retardant properties, flame retardant mechanism, and morphology.

## 2. Materials and Methods

### 2.1. Materials

DGEBA-type epoxy was kindly supplied by the Nan-Ya Plastics Corporation, Taipei City, Taiwan. 4,4′-Diaminodiphenylmethane (DDM) as a curing agent for epoxy and ammonium polyphosphate (APP) was purchased from San-Jin Chemicals Co. Kaohsiung, Taiwan. Fish scales (FSs) were obtained from the local market. Anhydrous stabilized tetrahydrofuran (THF) was obtained from Lancaster Co., Morecambe, Lancashire, UK.

### 2.2. Preparation of Epoxy/APP/FS Composites

**Scheme 1:**
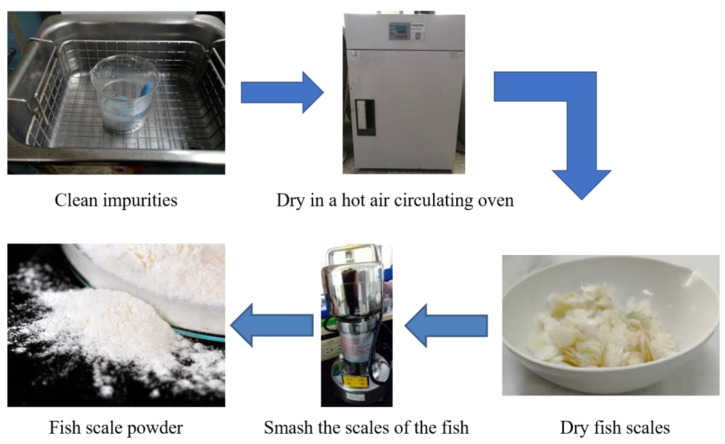
The preparation process of fish scale powder.

**Scheme 2:**
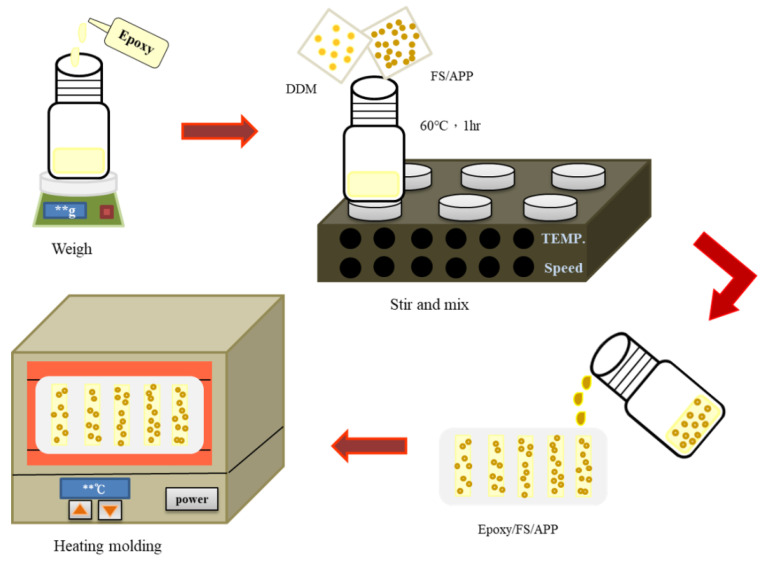
The preparation process of epoxy/FS/APP composites.

The FSs were sieved, cleansed with fresh water, and ultrasonicated to remove foreign matter. Subsequently, they were baked at 105 °C for 4 h and shredded into powder. After the FS powder was prepared, 2.83 g of APP and 5.67 g of FSs at a weight ratio of 1:2 were added to 60 mL of tetrahydrofuran and evenly mixed at 60 °C for 2.5 h. Then, 10 g of EP was evenly mixed into the APP/FS composite flame retardant, and 2.75 g of diaminodiphenylmethane (curing agent) was stirred into the mixture until it thickened, at which point the mixture was poured into a mold and left to set overnight. The next day, it was baked at 60 to 160 °C at increasing intervals of 20 °C every 2 h to complete the preparation of the EP/APP/FS composites. The detailed preparation processes are shown in Scheme 1 and Scheme 2.

### 2.3. Measurements

The samples were treated at 180 °C for 2 h and then ground into a fine powder. The thermal degradation of the composite was examined using a thermogravimetric analyzer (TGA) (Perkin Elmer TGA 7) from room temperature to 800 °C at a rate of 10 °C min^−1^ under an atmosphere of nitrogen. The measurements were made on 6–10 mg samples. Mass-loss/temperature curves were plotted. The LOI is defined as the minimum fraction of O_2_ in a mixture of O_2_ and N_2_ that will just support flaming combustion. The LOI test was performed according to the testing procedure of the ASTM D 2836 Oxygen Index Method, with a test specimen bar 7–15 cm long, 6.5 ± 0.5 mm wide, and 3.0 ± 0.5 mm thick. The sample bars were suspended vertically and ignited with a Bunsen burner. The flame was removed, and the timer was started. The concentration of oxygen was increased if the flame on the specimen was extinguished before burning for 3 min or burning away 5 cm of the bar. The oxygen content was adjusted until the limiting concentration was determined. The vertical burning test was performed inside a fume hood. Samples were held vertically with tongs at one end and burned from the free end. Samples were exposed to an ignition source for 10 s; then, they were allowed to burn above cotton wool until both the sample and cotton wool were extinguished. Observable parameters were recorded to assess fire retardancy. The UL 94 test classifies materials as V-0, V-1, and V-2 according to the time period needed before self-extinction and the occurrence of flaming dripping after removing the ignition source. V-0 is the most ambitious and desired classification. The morphology of the fractured surface of the composites was studied under a scanning electron microscope (SEM) (JEOL JSM 840A, Akishima, Japan). The distributions of Si atoms in the char were obtained from SEM EDX mapping (JEOL JSM 840A, Akishima, Japan). Raman spectra were recorded using a Lab Ram I confocal Raman spectrometer (Dilor, France). A He–Ne laser with a laser power of about 15 mW at the sample surface was utilized to provide an excitation wavelength of 632.8 nm. A holographic notch filter reflected the exciting line into an Olympus BX40 microscope, Tokyo, Japan.

### 2.4. Calculation of Experimental Data

The results are presented in the tables for the LOI test with the mean values and standard deviations. There are five sample quantities for each group for calculation. Statistical analysis was performed using one-way analysis of variance (ANOVA) for finding the differences within the sample group.

## 3. Results and Discussion

### 3.1. Thermal Properties

**Figure 1:**
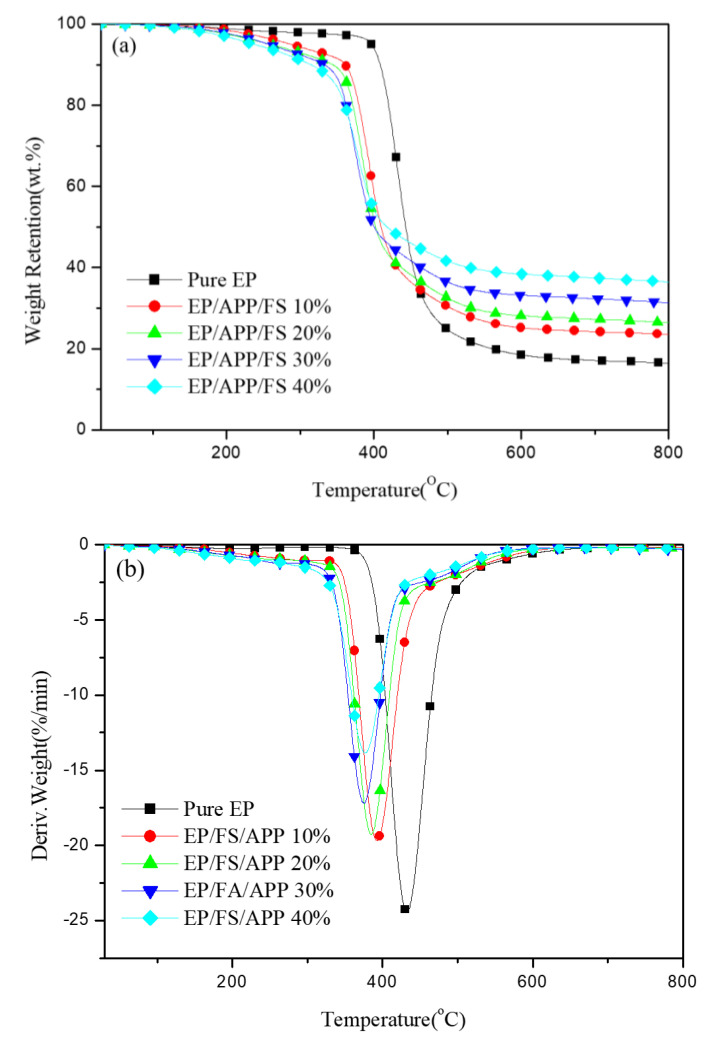
(**a**) TGA curves of epoxy and epoxy/APP/FS composites in N_2_. (**b**) Derivative curves of pure epoxy and epoxy/APP/FS composites in N_2_.

TGA involves the use of a microbalancer to record a specimen’s mass loss with temperature and determine the material’s thermal stability. In the TGA, different amounts of the APP/FS flame retardant were introduced to EP under a nitrogen environment at a heating rate of 20 °C/min to observe the mass loss of the mixture with temperature. Figure 1 and Table 1 present the results.

As shown in Figure 1a, the temperature at 5% mass loss (T_d5_) decreased considerably, from 397.1 °C (pristine EP) to 238.5 °C (EP/FS/APP 40%), a decrease of 60%. Likewise, char yield rose from 16.45 wt.% (pure EP) to 36.07 wt.% (EP/FS/APP 40%). The increase in char yield showed that phosphorus compositions in both APP and FS had a good catalytic capability to form char and could improve the stability of epoxy resin at high temperature [16,17,18,19,20,21,22,23]. FSs possessed hydroxyapatite, which was an inorganic component. This process contributed to the formation of a char layer that insulated the inner part of the specimen from heat, thus protecting the EP and increasing the overall thermal stability of the material [24,25].

Figure 1b presents the derivative thermogravimetric (DTG) curves, which demonstrate the trends of the materials reaching their maximum thermal degradation temperatures with time and temperature. According to Figure 1b, the maximum thermal degradation temperature decreased from 432.7 °C (pure EP) to 376.9 °C (EP/FS/APP 40%). This result can be explained by the presence of phosphorus in the flame retardant, which caused the material to break down at a lower temperature, contributing to the formation of a protective char layer through the mechanism of the condensed phase. The maximum thermal degradation rate decreased considerably, from −24.5 wt.%/min (pristine EP) to −13.9 wt.%/min (EP/FS/APP 40%), indicating a substantial improvement in EP’s thermal stability after the addition of the composite flame retardant.

Table 1 indicate that T_d5_ shifted leftward, the thermal degradation rate decreased, and CY increased substantially by 219% with the increase in flame retardant content. This result can be attributed to the presence of phosphorus, which induced the formation of phosphorus-containing char at a lower temperature, thereby protecting the matrix material. Hence, the composite flame retardant can effectively improve the thermal stability of polymeric composite materials.

**Figure 2:**
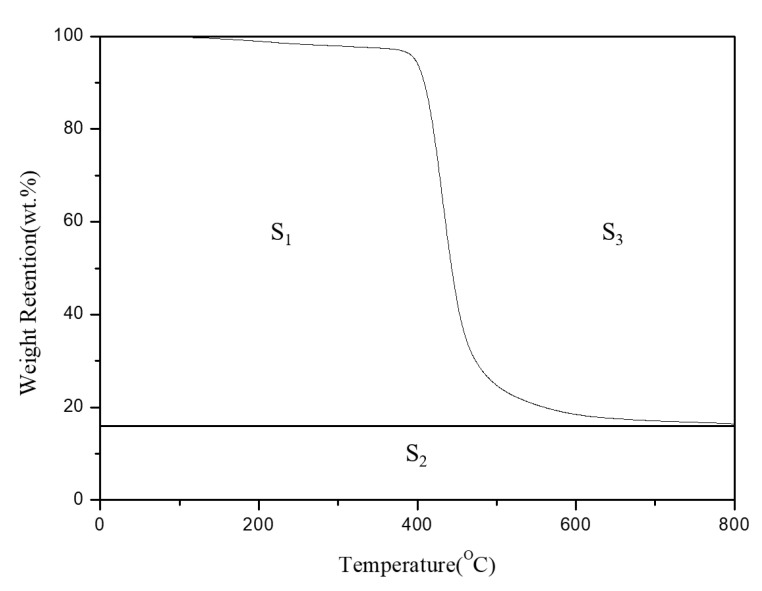
The TGA curve of the sample.

Integral procedural decomposition temperature (IPDT) is mainly used as an indicator of the thermal stability of polymeric materials [26,27]. IPDT can be obtained by integrating the area under the thermal decomposition curve and substituting it into an integration equation, as in Figure 2. A high IPDT indicates favorable thermal stability. IPDT can be affected by the initial thermal decomposition temperature and CY, which are also used as indicators of thermal stability. High initial thermal decomposition temperature and CY indicate high thermal stability and heat resistance; therefore, high IPDT. IPDT can be calculated by substituting T_i_, T_f_, S_1_, S_2_, and S_3_ into the following equations:

$$\mathrm{IPDT}({}^{\circ}C)=A^{*}\times K^{*}\times \left(\mathrm{Tf}-\mathrm{Ti}\right)+T_{i}.$$

$$A^{*}=\left(S_{1}+S_{2}\right)/\left(S_{1}+S_{2}+S_{3}\right)$$

$$K^{*}=\left(S_{1}+S_{2}\right)/S_{1}$$

$$\mathrm{Ti}=\mathrm{the}\;\mathrm{initial}\;\mathrm{experimental}\;\mathrm{temperature}\;\left(30\;{}^{\circ}C\right)$$

$$\mathrm{Tf}=\mathrm{the}\;\mathrm{final}\;\mathrm{experimental}\;\mathrm{temperature}\;\left(800\;{}^{\circ}C\right)$$

**Figure 3:**
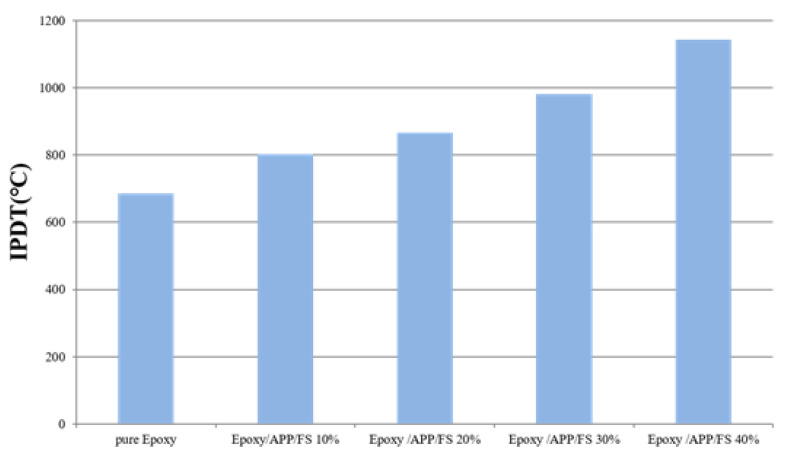
The IPDT data of pure epoxy and epoxy/APP/FS composites.

Table 1 presents the IPDTs of the EP/APP/FS composites. These IPDTs were 685.6 °C (pristine EP), 802.7 °C (EP/APP/FS 10%), 866.1 °C (EP/APP/FS 20%), 980.4 °C (EP/APP/FS 30%), and 1143.1 °C (EP/APP/FS 40%). The EP/APP/FS composites had IPDTs higher than that of pristine EP, increasing by 166.7%, confirming that the addition of the flame retardant improved the thermal stability of EP. The data in Table 1 and Figure 3 also support the TGA results.

The combustion of a polymeric material involves the burning of material with oxygen and the eventual formation of char residue and gas. However, before the polymeric material can begin to burn, an energy barrier must be overcome for the material to enter the thermal decomposition process. This energy barrier is the activation energy required for thermal decomposition. In this study, Ozawa’s method [28] was adopted to calculate the activation energy, the results of which are presented in Table 2. The R values of both pure EP and EP/APP/FS 40% exceeded 0.9, indicating excellent linearity. The activation energies of pure EP and EP/AP/FS 40% were 217 kJ/mole and 230 kJ/mole, respectively, suggesting that the addition of the flame retardant increased the activation energy required by the EP/APP/FS composites and improved their thermal stability. These findings also support the TGA results.

### 3.2. Flame Retardant Property

The UL94 flammability test involves the ignition of specimens of a specific size, whose burning durations are added to determine whether a material meets the UL94 standard and whether melt-dripping occurs (as evidenced by a cotton layer beneath the specimen being ignited by drips of molten material). On the basis of the test results, the flammability is rated as V-0, V-1, V-2, or fail [29,30].

A specimen is burnt for 10 s, after which the heat source is removed. The time from the removal of the heat source until the burning stops is recorded (t_1_). Subsequently, the specimen is burnt again for 10 s, and the time from the removal of the heat source until the burning stops is also recorded (t_2_).

V-0: If t_1_ + t_2_ < 10 s and no melt-dripping occurs, it is classified as a V-0 rating.

V-1: If t_1_ + t_2_ < 30 s and no melt-dripping occurs, it is classified as a V-1 rating.

V-2: If t_1_ + t_2_ < 30 s and melt-dripping occurs, it is classified as a V-2 rating.

Fail: If t_1_ + t_2_ > 30 s, it is classified as a fail rating.

As shown in Table 3, pristine EP was classified as a fail, whereas the addition of the composite flame retardant resulted in EP/APP/FS 40% being classified as a V-0 rating. The improvement in flame retardance is because APP decomposed into polyphosphoric acid, NH_3_ and H_2_O. Then, polyphosphoric acid furthermore dehydrated to generate P_4_O_10_ (or P_2_O_5_), which are inorganic gases. APP released PO· during its pyrolysis process to catch the free radicals from the polymer matrix. Moreover, the generation of phosphorus and polyphosphorus from the pyrolysis process of APP could catalyze the carbonyl group to form char during the cross-linking and esterification reactions. The overflow of NH_3_ and H_2_O resulting from APP decomposition caused the carbon layer to become an intumescent structure. Thus, an excellent residual char structure was formed on the surface of UPR composites [31]. 

LOI is widely used to test the flammability of materials through various oxygen concentrations. LOI involves increasing or decreasing the oxygen content of a confined space to determine the flammability of a material. The concentration of oxygen in the atmosphere is approximately 21%. A material can be classified as flammable, self-extinguishing, or fireproof depending on the test standards [32,33].

Flammable: LOI < 21%.

Self-extinguishing: 22% < LOI < 25%.

Fireproof: LOI > 26%.

LOI is calculated by using the following equation, where O_2_ and N_2_ represent the flow rates (mL/s) of oxygen and nitrogen, respectively:

$$\mathrm{LOI}=\frac{O_{2}}{O_{2}+N_{2}}\times 100\;\left(\%\right)$$

According to the results of the test, pristine EP had an LOI of 21%, marking it as a flammable polymer. After the addition of the flame retardant, however, the LOI of the EP/APP/FS 40% composite reached 36%, a 15% increase, suggesting that the composite had become a fireproof material, as shown in Table 3. This result can be attributed to the decomposition of APP, producing NH_3_ and polyacids. The flammable gas was diluted by the gas generated from decomposition, thereby slowing the combustion. The aromatic ring skeleton structure of EP began to break and interacted with polyacids to gradually form char residue. The newly formed carbon became a dense and strong carbon layer under the action of polyacids. The gas products produced caused the carbon layer to expand [34].

**Figure 4:**
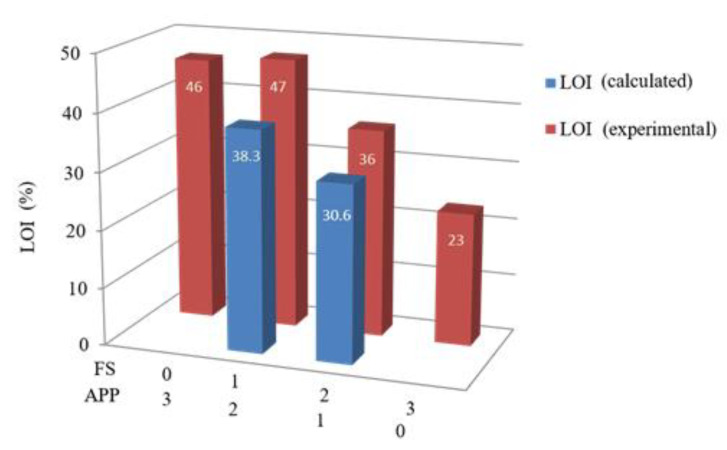
Comparison of calculated and experimental LOI of the composites.

As shown in Table 4 and Figure 4, the amount of flame retardant was fixed at 40 wt.%, and the proportions of FS and APP were changed to determine the synergy between the two components. When only one component was used in the flame retardant, the LOIs of the resultant composites were 46% (EP/APP) and 23% (EP/FS). When the ratio of FS:APP was 1:2 and 2:1, the LOIs were 47% and 36%, respectively. Figure 4 shows that when the FS:APP ratio was 1:2, the difference between the experimental and theoretical (calculated) LOI was 8.7, and when the FS:APP ratio was 2:1, the difference was 5.4. Thus, the experimental value was greater than the theoretical value in both cases, indicating that the commercial fire retardant and natural fire retardant synergize with each other. An FS:APP ratio of 2:1 was selected as the recommended ratio because of cost considerations in this study.

### 3.3. Morphological Properties

**Figure 5:**
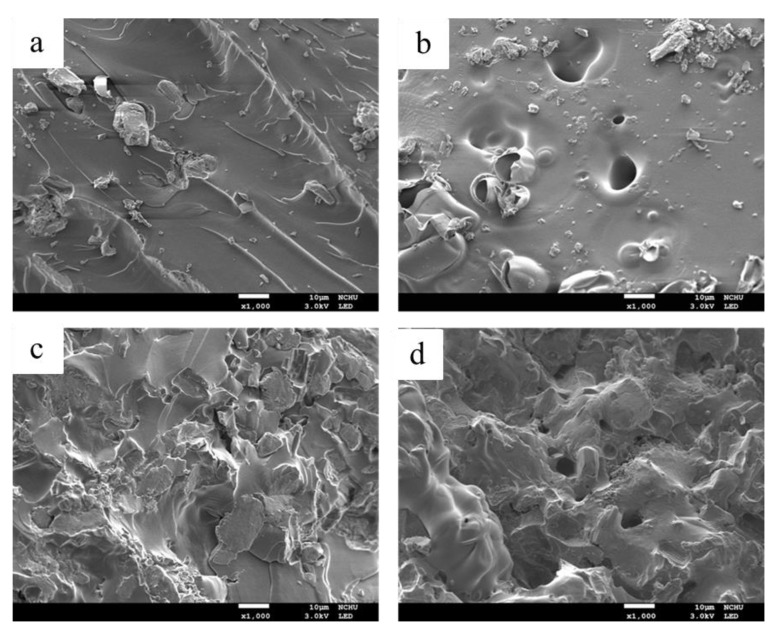
SEM micrographs of the composites: (**a**) epoxy/APP/FS 10% (before burning) (×1K); (**b**) epoxy/APP/FS 10% (after burning) (×1K); (**c**) epoxy/APP/FS 40% (before burning) (×1K); (**d**) epoxy/APP/FS 40% (after burning) (×1K).

Figure 5a,c present the surfaces of pure EP and EP/APP/FS 40% before burning. In Figure 5a, the particles visible on the surface are grains of APP/FS that were added. In Figure 5c, the presence of numerous particles and the irregular surface structure are the result of the addition of a large amount of flame retardant. Figure 5b presents EP/APP/FS 10% after burning. A light layer of char was observed, and the holes and pits were formed by the gas released by APP when burning. These holes and pits compromised the protection on the polymer matrix material because they allowed flames to pass through and reach the base material. For this reason, the performance of this composite was deemed unsatisfactory. Figure 5d presents EP/APP/FS 40% after burning. A dense char layer on the surface barred the transmission of oxygen and heat, thereby raising the thermal stability of the composite. The composites formed an intumescent morphology after burning because APP as gas sources released inflammable gases during combustion and expended the char layer. At the same time, FS was used as a char source to be dehydrated and carbonized, eventually forming intumescent and dense char residuals [35].

**Figure 6:**
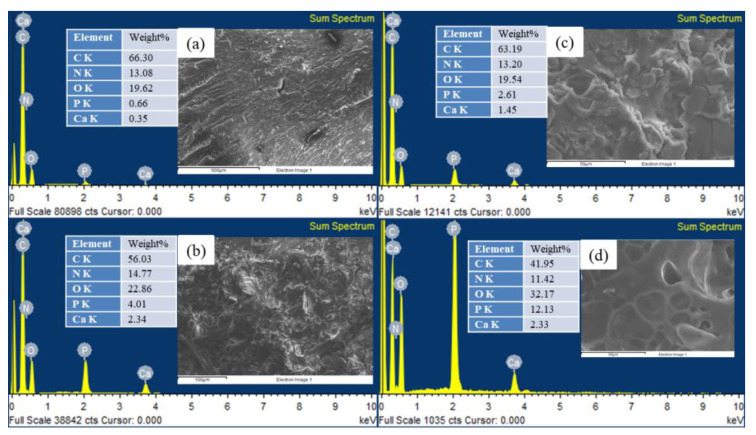
EDS of (**a**) epoxy/APP/FS 10% (before burning); (**b**) epoxy/APP/FS 10% (after burning); (**c**) epoxy/APP/FS 40% (before burning); and (**d**) epoxy/APP/FS 40% (after burning).

Figure 6 and Table 5 present the EDS results of the EP/APP/FS composites containing different proportions of the APP/FS flame retardant. Before being burned, EP/APP/FS 10% contained C, O, N, P, and Ca with weight percentages of 66.3 wt.%, 13.08 wt.%, 19.62 wt.%, 0.66 wt.%, and 0.35 wt.%, respectively. After raising the flame retardant content to 40%, the contents of N, P, and Ca in EP/APP/FS 40% increased. This was caused by the addition of the flame retardants, which contained these elements. Specifically, P increased from 0.66 wt.% to 2.61 wt.%, and Ca rose from 0.35 wt.% to 1.45 wt.%. When burned, the P in EP/APP/FS 40% dehydrated the material, forming a phosphate-based char layer that raised the weight percentage of P to 12.13 wt.% after the material was burned. This indicates that in the burning process, the presence of P increases CY, thereby facilitating the formation of a char layer that covers the surface of a matrix material and prevents it from being burned further.

### 3.4. Char Analysis

This study used Raman spectroscopy to analyze the char of the EP/APP/FS composites, which were left in a high-temperature environment (600 °C) for 1 and 5 min. The Raman spectrum was observed for changes in the disorder (D)-band and graphitic (G)-band [36,37]. The D-band, which is located at 1350 cm^−1^, represents an irregular sp^3^ structure formed by linear carbon chains. After being burned, it becomes char, turning from the D-band into the G-band. The G-band is located at 1580 cm^−1^ and represents an sp^2^ structure formed by hexagonal carbon rings, the structure of graphite. The presence of a larger number of G-bands indicates an abundance of char, which is a favorable carbonized state, because char is mainly composed of hexagonal carbon rings. Dividing the area of the D-band by that of the G-band produces a D:G ratio; smaller ratios indicate a larger abundance of graphite, which indicates that more char that is formed, as shown in Table 6.

**Figure 7:**
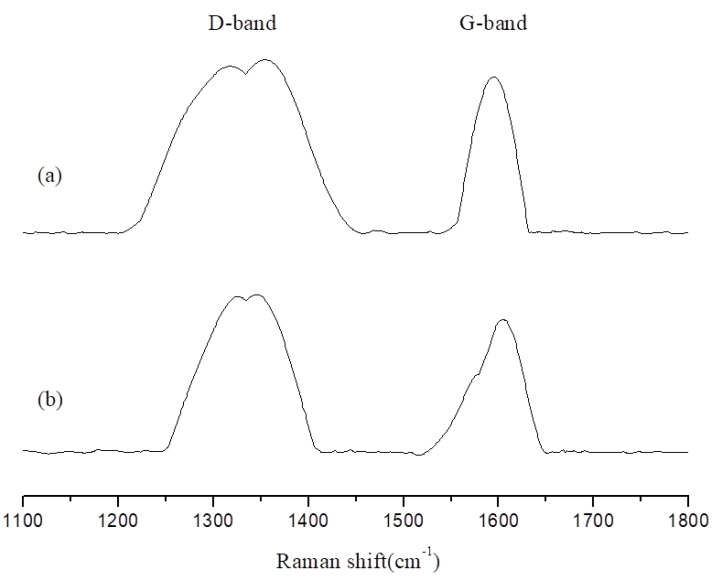
The Raman spectra of char products from epoxy/APP/FS 10% at 800 °C: (**a**) 1 min; (**b**) 5 min.

**Figure 8:**
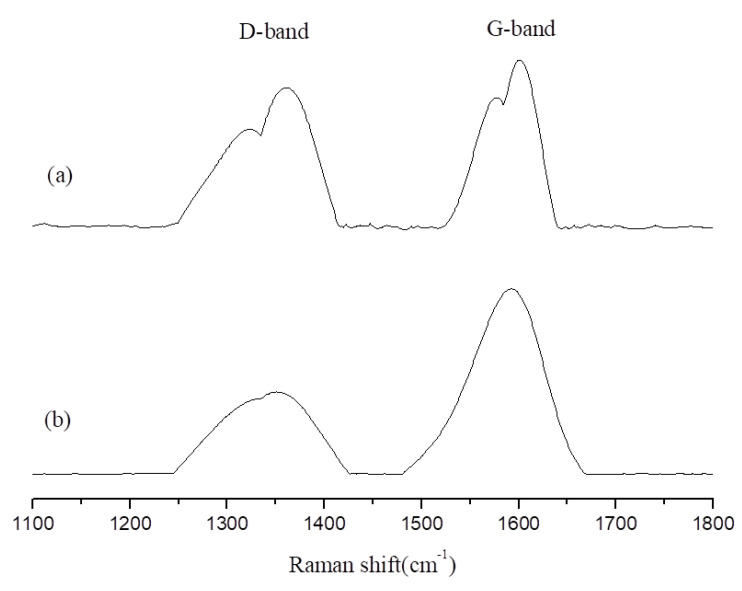
The Raman spectra of char products from epoxy/APP/FS 40% at 800 °C: (**a**) 1 min; (**b**) 5 min.

Figure 7 and Figure 8 present the changes in the D-band and G-band of EP/APP/FS 10% and EP/APP/FS 40%. According to the analytic results presented in Figure 7, both low and high concentrations increased the amount of char. This indicates that the addition of the composite flame retardant effectively increased the amount of char formed in the burning process, which made the material more difficult to burn, and that the flame retardant effect was greater at a higher concentration.

## 4. Conclusions

This study addressed the problems that EP that is highly flammable and that it does not self-extinguish once on fire. The solution proposed in this study was the introduction of a composite flame retardant comprising both APP and FSs, which was proven to lower the flammability of the polymeric matrix material and make it more difficult to burn. The inclusion of FS can lower the consumption of APP and is also a suitable use for a waste material. Therefore, it is a cost-efficient and environmentally friendly approach that can serve as an element in the circular economy. When the proportion of the APP/FS flame retardant in EP was raised to 40%, it effectively raised CY to 36.07 wt.%, IPDT to 1143.1 °C, UL94 classification to V-0, and LOI to 36%, thus turning EP from a flammable material into a fireproof material. These results indicate that adding the APP/FS flame retardant to EP to form an EP/APP/FS composite increases the thermal stability of the material and considerably reduces its flammability.

The addition of the dual flame retardant increased the activation energy of thermal degradation required by the EP/APP/FS composites and improved their thermal stability. Excellent thermal stability promotes the flame retardance of a material. The more flame retardant is added, the better the flame retardant properties will be. The flame retardant in this study belonged to the additive type of flame retardant. When it was added to 30%, its LOI value reached 27%, indicating the flame resistance level, mainly due to APP. There is a synergistic effect with fish scales: an FS/APP ratio of 2:1 was the most cost-effective and effective formula for this study.

Fish scales are originally fishery wastes. Through this research, they can be used together with commercial flame retardants to improve the flame retardance of epoxy resins, and greatly reduce the use of commercial flame retardants, which can solve the problem of environmental pollution and increase the value of fish scales.

## Figures and Tables

**Table 1:** Thermal properties of epoxy/APP/FS composites.

Sample NO.	^a^ T_d5_ (°C)	^b^ T_max_ (°C)	^c^ R_max_ (wt.%/min)	IPDT (°C)	C.Y (wt.%)
Pure epoxy	397.1	432.7	−24.5	685.6	16.45
EP/APP/FS 10%	288.7	392.4	−19.6	802.7	23.56
EP/APP/FS 20%	260.4	384.9	−19.3	866.1	26.44
EP/APP/FS 30%	258.2	378.7	−17.2	980.4	30.80
EP/APP/FS 40%	238.5	376.9	−13.9	1143.1	36.07

^a^ T_d5_ is the temperature when the weight loss of sample reached 5%. ^b^ T_max_ corresponds to the temperature of the maximum degradation rate. ^c^ R_max_ corresponds to the maximum thermal degradation rate.

**Table 2:** The calculated activation energy of thermal degradation with various conversions by Ozawa’s method.

α	EP		EP/FS/APP 40%	
	E (kJ/mole)	R Value	E (kJ/mole)	R Value
0.1	241	0.935	93	0.901
0.2	230	0.966	202	0.938
0.3	216	0.984	242	0.920
0.4	195	0.992	228	0.951
0.5	183	0.998	202	0.974
0.6	184	0.999	186	0.985
0.7	188	0.999	200	0.996
0.8	218	0.999	419	0.993
0.9	299	0.990	304	0.989
∆E(av)	217		230	

**Table 3:** The flame retardance of epoxy/APP/FS composites by UL-94 and LOI values.

Sample	UL-94		LOI (%)	∆LOI (%)
	Ranking	Dripping		
Pure epoxy	Fail	NO	21.2 ± 0.2	0
EP/APP/FS 10%	Fail	NO	21.3 ± 0.3	0.1
EP/APP/FS 20%	Fail	NO	23.1 ± 0.2	1.9
EP/APP/FS 30%	V-0	NO	27.2 ± 0.3	6.0
EP/APP/FS 40%	V-0	NO	36.2 ± 0.2	15.0

**Table 4:** Comparison of calculated and experimental LOI data of the composites.

Sample No.	FS:APP	LOI (%)
EP/APP 40% (experimental)	0:3	46.2 ± 0.1
EP/APP/FS 40% (experimental)	1:2	47.3 ± 0.3
EP/APP/FS 40% (experimental)	2:1	36.2 ± 0.2
EP/FS 40% (experimental)	3:0	23.3 ± 0.2
EP/APP/FS 40% (calculated)	1:2	38.3 ± 0.2
EP/APP/FS 40% (calculated)	2:1	30.6 ± 0.3

**Table 5:** EDS for epoxy/APP/FS composites.

Sample No.	Elements				
	C (wt.%)	N (wt.%)	O (wt.%)	P (wt.%)	Ca (wt.%)
EP/APP/FS 10% (before burning)	66.3	13.08	19.62	0.66	0.35
EP/APP/FS 10% (after burning)	56.03	14.77	22.86	4.01	2.34
EP/APP/FS 40% (before burning)	63.19	13.20	19.54	2.61	1.45
EP/APP/FS 40% (after burning)	41.95	11.42	32.17	12.13	2.33

**Table 6:** The area ratio of Raman shift from the char of epoxy/APP/FS composites.

Sample No.		D-band	G-band	D/G
		1350 cm^−1^	1850 cm^−1^	
EP/APP/FS 10%	1 min	723,254	220,353	3.282
	5 min	106,075	106,075	2.019
EP/APP/FS 40%	1 min	514,002	414,064	1.241
	5 min	176,812	322,428	0.548

## Data Availability

The data presented in this study are available on request from the corresponding author.
